# Public reporting on pharmaceutical industry-led access programs: alignment with the WHO medicine programs evaluation checklist

**DOI:** 10.1186/s40545-020-0204-z

**Published:** 2020-03-26

**Authors:** Chukwuemeka A. Umeh, Peter C. Rockers, Richard Laing, Ovi Wagh, Veronika J. Wirtz

**Affiliations:** 1 Department of Global Health, Boston University School of Public Health, 801 Massachusetts Avenue, Boston, MA 02118 USA; 2 Faculty of Community and Health Sciences, University of Western Cape, Cape Town, South Africa

**Keywords:** Medicines, Access, Industry, Private sector, Transparency, Accountability

## Abstract

**Background:**

There has been increased demand for greater public accountability and transparency of private sector-led global health partnership programs. This study critically reviews and pilot tests the World Health Organization (WHO) medicine program checklist as a framework for public reporting and assessing of programs.

**Methods:**

We reviewed each question on the WHO checklist for clarity and usability. Next, we pilot tested the subset of checklist questions focused on program assessment. We extracted and analyzed publicly available information on one randomly selected program from each of the 20 largest research-based biopharmaceutical companies. For each program, we assessed whether publicly available information allowed for an assessment of each relevant question in the checklist.

**Results:**

Checklist questions fit in four main categories: [1] national health and development plans, needs, capacity, laws and policies; [2] financial, performance, and public accountability; [3] risk management and mitigation strategies; and [4] long-term sustainability. Nearly all (21 of 22) questions in the checklist require information best provided by companies; one question requires information best provided by governments.

Programs frequently reported on the public health needs of their programs (100%), program objectives and activities (100%) and the actual or expected program outputs (95%). There was less information on program alignment with country plans and capacity (50%), detailed program monitoring and evaluation plan (20%), risks mitigation strategies (5%), program needs assessment (5%), and additional resources required from or contributed by government (0%).

**Conclusion:**

The WHO checklist of key considerations for evaluating proposals for access to medicine programs could be a useful framework for public reporting of program information as most of checklist questions ask for data that should be available to those leading the program. Further revisions of the WHO checklist will help refine it to improve clarity and content validity.

## Background

The number and scope of public health programs implemented by biopharmaceutical companies to promote access to care and medicines for people in low- and middle-income countries has increased in recent years [1]. This increase has triggered wider discussions on the gaps in program performance monitoring and evaluation [2, 3], public policies on the roles of the private sector [4], and public accountability including the absence of an assessment tool to guide governments in their review of program development, implementation and regulation [5]. While private sector companies are traditionally accountable to shareholders and investors, and public sector organizations are accountable to the political structures, there has been lack of public accountability and transparency of global health public-private partnership programs [6, 7]. The World Health Organization has defined accountability within health systems as “a relationship between a duty holder and a person or organization to whom a duty is owed. It describes the capacity to demand that a person or organization give reasons to justify their behavior and the capacity to impose a sanction if they fail to give reasons, or if their performance is poor.” [8] Transparency is often regarded as a prerequisite for accountability [9].

Many programs implemented by biopharmaceutical companies seem to appreciate the importance of public transparency and accountability by hosting a website and publishing annual reports; however, these usually contain little information on program governance, needs assessment and alignment with country plans and capacity, detailed program plan, program impact and sustainability plans [7]. When programs are open and forthcoming with information on all aspects of the program, this allows program assessment by stakeholders which helps to build public trust in the program [10].

In 2017, the World Health Organization (WHO) published a checklist of key considerations that governments should consider when evaluating proposals for access to medicine programs from medicines and medical device companies (hereafter referred to as the "WHO checklist") [5]. The motivation for such a checklist was the need for guidance of how to evaluate whether a program aligns with national health and development plans, needs, capacity, laws and policies; has mechanisms to ensure financial, performance, and public accountability; has risk management and mitigation strategies and has transitioning plans for long-term sustainability.

Although the WHO checklist was developed to provide a uniform framework through which programs can provide information to governments, it could also serve as a useful framework for public reporting of program information in the absence of any other reporting framework. It is important that the public is not only informed of the programs successes and lessons learned as is currently the case but has access to the evidence and assumptions used to inform program planning such as needs assessment documents, detailed program plan, monitoring and evaluation plan, sustainability plans and potential conflict of interest [10].

The objective of this study is to test the feasibility of using the WHO checklist to assess key considerations for evaluating proposals for access to medicine programs as a framework for reporting program information. The WHO checklist was designed to assess information submitted to government by companies and from our perspective all such program information should be in the public sphere to ensure accountability and transparency [11]. Based on this perspective, we reviewed publicly available program information and made suggestions on which program information responds to specific questions in the checklist and how the checklist could be modified to serve as a framework for public program reporting.

## Methods

For the purpose of this study we defined access programs led by medicines and medical device companies as those that are designed and co-financed by pharmaceutical companies and that they take responsibility and credit for them (Rockers et al., 2018). Depending on the industry-led program it could be implemented in collaboration with the government, other pharmaceutical companies or NGOs. The program can be philanthropic or has shared business and social goals.

We used a two-stage process in conducting this study. In the first stage, we reviewed each question on the WHO checklist for clarity and took note of double- or multi-barreled questions (questions that had two or more concepts in a single question). We also critically reviewed the checklist to determine which questions require information best provided by companies versus government when using the checklist as a framework for public reporting of program information. We then described each question on the checklist and provided examples of program information that may or may not be good responses to each question on the checklist.

In the second stage, we extracted and analyzed publicly available information of 20 biopharmaceutical industry led programs, with one program randomly selected from each of the 20 largest research-based pharmaceutical companies [12]. Our program sample frame was each company’s list of global health partnership programs in the International Federation of Pharmaceutical Manufacturers and Associations (IFPMA) World Health Partnerships Directory [13], which to our knowledge has the most comprehensive listing of pharmaceutical industry-led health programs. For the two companies, Gilead and Novo Nordisk, two recent IFPMA members with no single company programs listed on the IFPMA directory as at the time of our study, we randomly selected an ongoing program from the company’s 2017 annual report (Gilead) and company’s website (Novo Nordisk).

For the programs selected, two researchers independently searched online for program information and reviewed program webpages, press releases, reports, and peer reviewed articles. They also reviewed program information reported in the IFPMA Health Partnerships Directory [13], Access Observatory [11] and Access Accelerated Open Platform [14]. The Access Accelerated Open Platform is a directory of Access Accelerated initiative programs, comprising of about 90 programs as of December 2018 [14]. Access Accelerated is a partnership of more than 20 global research and development-based biopharmaceutical companies, working with partners including the World Bank and the City Cancer Challenge that seeks to reduce barriers to prevention, treatment and care for patients with non-communicable diseases in low- and middle-income countries.

Access Observatory is a public reporting platform with the aim to improve access to disease prevention and treatment services in low- and middle-income countries, including those designed and implemented by public and non-profit organizations. The Access Observatory was created in 2017 to report and evaluate the Access Accelerated programs [11].

For each program, the researchers independently noted which of the program’s publicly available information responds to the questions on the WHO checklist. We considered programs that provided information on any of the constructs in the double- or multi-barreled questions on the WHO checklist, as having responded to that question. Furthermore, we interpreted the question “Due diligence on industry partner has been conducted” in Section 3 on Strong Risk Management and Mitigation Strategies to mean that companies have provided information on how they assessed and selected their funding or implementing partners. In our analysis, we combined the following two questions in the WHO checklist, “The initiative can be implemented under existing legislation and it adheres to national regulations, procurement procedures, treatment guidelines, and standards of care, quality and safety requirements, remuneration scales and hiring practices” and “The program is suitable for the existing infrastructure, capacity, environment and local context”. Only if both statements applied, did we check this combined item.

The researchers met to reach a consensus if there was any disparity in the information they independently extracted. Thereafter, we calculated the proportion of programs with publicly available information on each of the questions on the checklist.

## Results

The WHO checklist has four domains and 23 questions of key considerations to be taken into account by companies when providing information on access to medicine programs. Based on our analysis, information on 22 out of 23 questions could easily be provided by company programs when using the checklist as a framework for public reporting of program information. Governments are in a better position to provide information on one of the questions “There is sufficient support for the program amongst political parties, unions, and civil society organizations”. Two of the remaining 22 questions were combined into one, leaving 21 in the final checklist. Six of the questions (27%) contained two or more related constructs in one question (Table 1).

**Table 1:** Description of program information that would fulfil the requirements of the corresponding checklist question

	WHO checklist	Description of program information from companies that align with the checklist
**Alignment with countries’ national health and development plans, needs, capacity, laws and policies**		
1	The program serves a public health need.	A description of the health problem the program is trying to solve.
2	The policy objective is clear.	Program objectives are clearly stated.
3	The program aligns with health strategic plans and the general development agenda.^a^	A description of how the program aligns with the country’s health strategic plan and/or development agenda. For multi-country programs, a description of how the program aligns with the Sustainable Development Goals or World Health Organization health goals might meet this requirement.
4	The program is suitable for the existing infrastructure, capacity, environment and local context.^a b^	A description of the design or implementation of programs in accordance with country laws, policies and practices might meet this requirement, e.g. treating patients using national treatment guidelines.
		Just mentioning that the government and local stakeholders made inputs to the program design might not meet this requirement.
5	Additional government resources (infrastructure, human resources or funding) that are required have been identified and are available.^a^	A description of additional resources that are required from government e.g. human resources and infrastructure.
6	The program does not divert resources away from other public health priorities.	Information on how the additional resources that might be required from government will not adversely affect other public health priorities.
7	The program has been compared to other approaches/programs/ programmes and has been found to be the most suitable.^a^	A formal needs assessment should provide information on the suitability of the program.
**Strong mechanisms to ensure financial, performance, and public accountability**		
1	Roles and responsibilities for all stakeholders involved are clear.	A description of the roles and responsibilities of all named stakeholders meets this requirement.
2	The mechanisms for how the program will be carried out are clear.	A description of program activities might meet this requirement.
		However, a more detailed work plan showing the program activities, and the responsible party and timeline for each activity will be ideal.
3	Those responsible for overseeing and monitoring the program have been identified.	A mention of the party responsible for monitoring or evaluating the program might meet this requirement.
4	The process for monitoring and evaluation has been established.	A description of the process for monitoring and evaluation of the program. This may include a description of the performance indicators and data collection and processing strategies.
5	Allocation, disbursement and utilization of financial resources have been defined.^a^	The program should provide information on the allocation, disbursement and utilization of financial resources. Reporting the amount that a program has spent or plan to spend might meet this requirement.
6	Performance targets, outputs and results are defined.^a^	Reporting the performance targets and/or program outputs/outcomes meets this requirement e.g. number of persons the program plans to treat. For programs that are already being implemented, reporting both the performance target and the actual outputs and results is ideal.
7	There is sufficient support for the program amongst political parties, unions, and civil society organizations.	Government should provide this information.
8	Measures to disclose information to the public, including procurement information, contractual obligations, evaluation criteria, progress reports, fund flows, commitments and timelines have been established.	A description of the plan to make program information publicly available.
		Providing program information through webpages, program reports, annual company reports, etc. might meet this requirement.
**Strong risk management and mitigation strategies**		
1	Risks have been identified.	A description of the potential strategic, implementation, and sustainability risks, harm or unintended consequences might meet this requirement.
2	Mitigation strategies for each risk, have been developed.	A description of how to mitigate any potential strategic, implementation, and sustainability risks, harm or unintended consequences might meet this requirement.
3	Due diligence on industry partner has been conducted (including financial, managerial and implementation capacity assessments).	Company provides information on how it assessed and selected its partners.
4	Potential conflicts of interest have been identified.	A description of any potential conflict of interest meets this requirement such as potential conflicts between business interests and social interests (program goals).
**Clear transitioning plans for long-term sustainability**		
1	The program is or will be integrated into the health system.	A description of how the program will be integrated into the health system meets this requirement e.g. incorporating the program’s training curriculum for midwives into the national training curriculum. A program that is supporting an existing government program might meet this requirement.
2	A clear transition plan for when the program ends has been developed.	A description of how the program will transition to the local community/government or made self-sustaining when the funding has ended meets this requirement. For example, having training participants pay a training fee to sustain the program.
3	A strategy to ensure sustainability of health gains has been developed.	A description of how the program will ensure sustainability of the health gains meets this requirement e.g. training local health providers to train other providers who will continue to provide care after the program has ended.
		A program that ensures local ownership of the program might meet this requirement.
		A pricing scheme where patients pay part of the cost of the medicine might meet this requirement.
		A licensing agreement that leads to lower prices for patients might meet this requirement.

^a^Double- or multi-barreled questions (questions that had two or more concepts in a single question)

^b^ We combined two questions: “The initiative can be implemented under existing legislation and it adheres to national regulations, procurement procedures, treatment guidelines, and standards of care, quality and safety requirements, remuneration scales and hiring practices” and “The program is suitable for the existing infrastructure, capacity, environment and local context” into one question because of their similarity. Only if both statements applied, we checked this item

Note: “The policy objective is clear”: for the purposes of this study, we considered program that provided information on their program objectives as having answered the question

Pilot testing the checklist using information from 20 programs showed that programs were more likely to provide information on mechanisms to ensure financial, performance, and public accountability (on average, 11 programs answered each of the eight questions in this domain; 55%) (Table 2). This is followed by information on how the programs align with the countries’ national health and development plans, needs, capacity, laws and policies (on average, eight programs answered each of the seven questions in this domain; 40%) and transitioning plans for long-term sustainability (on average, six programs answered each of the three questions in this domain; 30%). Programs were least likely to provide information on risk management and mitigation strategies (on average, one program answered each of the three questions in this domain; 4%).

**Table 2:** Number of programs that provide information on each checklist question

WHO checklist	Total	Percent (*N* = 20)
**Alignment with countries’ national health and development plans, needs, capacity, laws and policies**	**7.86**	**39.29%**
The program serves a public health need.	20	100%
The policy objective is clear.	20	100%
The program aligns with health strategic plans and the general development agenda.	10	50%
The program is suitable for the existing infrastructure, capacity, environment and local context.	4	20%
Additional government resources (infrastructure, human resources or funding) that are required have been identified and are available.	0	0%
The program does not divert resources away from other public health priorities.	0	0%
The program has been compared to other approaches/programs/ programmes and has been found to be the most suitable.	1	5%
**Strong mechanisms to ensure financial, performance, and public accountability**	**10.75**	**53.75%**
Roles and responsibilities for all stakeholders involved are clear.	7	35%
The mechanisms for how the program will be carried out are clear.	19	95%
Those responsible for overseeing and monitoring the program have been identified.	10	50%
The process for monitoring and evaluation has been established.	4	20%
Allocation, disbursement and utilization of financial resources have been defined.	7	35%
Performance targets, outputs and results are defined.	19	95%
There is sufficient support for the program amongst political parties, unions, and civil society organizations.	0	0%
Measures to disclose information to the public, including procurement information, contractual obligations, evaluation criteria, progress reports, fund flows, commitments and timelines have been established.	20	100%
**Strong risk management and mitigation strategies**	**0.75**	**3.75%**
Risks have been identified.	1	5%
Mitigation strategies for each risk, have been developed.	1	5%
Due diligence on industry partner has been conducted (including financial, managerial and implementation capacity assessments).	1	5%
Potential conflicts of interest have been identified.	0	0%
**Clear transitioning plans for long-term sustainability**	**6**	**30%**
The program is or will be integrated into the health system.	7	35%
A clear transition plan for when the program ends has been developed.	0	0%
A strategy to ensure sustainability of health gains has been developed.	11	55%

For the national health and development plans, needs, capacity, laws and policies domain, programs clearly stated their program objectives (*n* = 20/20; 100%), described how their programs serve a public health need (*n* = 20/20; 100%) and how their programs align with health strategic plans and general development agenda (*n* = 10/20; 50%). Only a few programs reported on how their programs are suitable for existing infrastructure, capacity, environment and local context (*n* = 4/20; 20%). One program (a HIV screening and linkage to care program) reported on how needs assessment was used to inform program design. None of the programs provided information on additional government resources required for their programs and none provided information on how and/or why their programs divert or do not divert resources away from other public health priorities.

For the financial, performance, and public accountability domain, programs provided information on how the programs will be implemented (*n* = 19/20; 95%), program performance targets or outputs (*n* = 19/20; 95%), parties responsible for overseeing and monitoring the programs (*n* = 10/20; 50%) and the roles and responsibilities of all identified stakeholders (*n* = 7/20; 35%). There was limited information on the financial resources budgeted or used for the project (*n* = 7/20; 35%) and the process for monitoring and evaluation – data collection, processing, and validation (*n* = 4/20; 20%).

For the risk management and mitigation strategies domain, only one program (a licensing agreement program) identified the risks and has risk mitigation strategies and one program reported on how their partners were selected (a HIV screening and linkage to care program). None of the programs reported identifying potential conflicts of interest.

For the transitioning plan for long-term sustainability domain, more than half of the programs reported on a strategy or designed their programs in such a way as to ensure sustainability of health gains (55%) while 35% of programs were part of existing government program or will be integrated into the health system. None of the programs reported developing a clear transition plan for when the program ends.

## Discussion

There is increased demand for greater public accountability and transparency of global health partnership programs. The WHO checklist was developed as a framework to assess key considerations for evaluating proposals for access to medicine programs. To our knowledge this is the first time the checklist has been pilot tested to study its feasibility as a framework for public reporting of program information..

Our study contributes to existing knowledge in several aspects. First, our results demonstrate the usefulness of the checklist in systematically assessing programs. Most of checklist questions ask for information that should be available to those leading or reporting about the program. Setting standards in industry-led social program reporting is relevant to increase transparency and enable accountability [15]. Although progress has been made in reporting on environmental social corporate responsibility aspects, there is a large body of literature on corporate social responsibility that shows that industry led programs lack reporting standards [16, 17]. However, there are very few publications discussing the expectations for pharmaceutical-industry led access programs and their compliance with reporting standards comparable to other industries [18].

Second, the piloting of the checklist showed the existing gaps in the public reporting of program information and the areas on which companies might need to focus more attention. We used publicly available program data because from our perspective, program information that companies submit to government should be in the public sphere to ensure accountability and transparency. In general, most programs were clear on the public health needs of their programs, program objectives and activities and the actual or expected program outputs. However, there was little information on additional resources required from or contributed by government and other stakeholders; program needs assessment, program alignment with country plans and capacity, detailed program monitoring and evaluation plan, risks mitigation strategies and sustainability plans. This finding agrees with an earlier observation of public-private partnerships by Buse et al. [7] Specifically, we noted that most programs did not report their risk management and mitigation strategies. Though the reason for this finding is not clear, it could be that the companies did not realize the importance of such reporting or did not conduct systematic program risk assessment and so do not have any information to provide. Assessing potential program initiation, implementation and sustainability risk, including financial sustainability risk is important, as the role of sustainable funding in program sustainability cannot be overemphasized [19, 20]. In addition, it is important to assess the capacity of the implementing partners to implement the programs as studies have linked program sustainability to the organizational capacity of the implementing organization [19, 21].

Moreover, companies are currently reporting their program objectives and the public health needs that their programs meet. However, there is paucity of reporting program needs assessments, as many of the programs were silent on how or whether the needs assessment influenced program design. Conducting a needs assessment before the start of a program is invaluable as it helps organizations to use their limited resources to help communities in the most efficient ways [22]. It is not just important for companies to do a needs’ assessment; it is also important that the public are made aware of the evidence and assumptions used to inform program planning as this will help build public trust in the program [10]. In addition, all the programs were silent on any additional government resources needed for their programs. There are concerns that cost of programs to recipient countries such as the cost of providing a distribution network for donated medicines and training health workers, might lead to diverting domestic resources from national priorities, which might worsen the inequalities affecting vulnerable groups [23–25]. To ensure transparency, it is important that programs report the additional resources required from government or any of its stakeholders. This will ensure that the contributions of all stakeholders are fully acknowledged.

Furthermore, apart from one donation program, all the programs clearly reported on how they are implementing their programs. The reason for the lack of information on the donation program, which is part of a Ministry of Health Pharmaceutical Assistance Program, is not clear. In addition, all programs, except for one new program that is still in the need’s assessment stage, reported their performance target or program output. However, it appears programs are behind in setting up systems for program evaluation, as there is paucity of information on the process for program monitoring and evaluation. This might not be limited to programs in this study, as earlier studies of access to medicine programs have shown few independent and rigorous evaluations of program impact [1].

The transitioning plans for long-term sustainability domain has three questions, which appears to follow Shediac-Rizkallah and Bone three indicators of sustainability [26]. It measured sustainability at: [1] the individual level - a strategy to ensure sustainability of health gains [2]; the organization level - a clear transition plan for when the program ends; and [3] the community level - the program is or will be integrated into the health system. While none of the programs reported a clear transition plan for when the program ends, about a third of the programs were part of existing government programs or will be integrated into the health system. An encouraging finding was that more than 50% of programs designed their programs in ways that will ensure sustainable health gains for individuals such as training local providers to train other providers who will continue to provide care after the program has ended. Overall, though the WHO checklist was initially prepared for governments to evaluate proposals for access to medicine programs from medicines and medical device companies, it could also be a framework for the public reporting of program information by companies. The checklist is setting standards for reporting that enable governments, civil society, professional organizations and other advocacy groups to refer to them and ensure that the information is provided before permission of program operation is granted.

There are some areas in which the WHO checklist could be strengthened. We noted that the WHO checklist does not ask any question on how programs address social inequity. Information on how public-private partnerships have affected health equity is scarce and programs ability to address social inequity should be an important consideration in access programs [6]. Social inequities exist between and within countries with those in disadvantaged groups having worse health outcomes [27]. The 2015 United Nations sustainable development goals highlights the importance of reducing inequality within countries as a key step to ensuring sustainable development [28]. Reporting how access programs plan to address inequity will make it easier to assess what equity targets are set and who should be held accountable for achieving those targets, if any. In addition, the checklist does not ask whether the program has been evaluated in terms of its performance. Information on program performance in other settings could provide governments and other stakeholders with valuable insights about the expected effects of the program in their country. Whether an impact evaluation (the extent to which the program’s achievements can be attributed to the program interventions) or a process evaluation (the extent to which the program has been implemented as planned) is appropriate depends on a number of factors. An impact evaluation may be justified for programs that are expected to be implemented in many countries and cover a substantial number of people such as the Novartis Access program which was initially planned to be implemented in 30 countries [29]. Smaller programs covering fewer people or programs using interventions that are well evaluated may not require an impact evaluation depending on the setting in which they are implemented. Adding the requirement to report on any process and impact evaluation would enhance the value of the checklist.

Finally, double or multi-barreled questions can make respondents uncertain on how to respond to such questions [30, 31]. One way to deal with those questions will be to split them into separate questions. For example, the question “The program aligns with health strategic plans ***and*** the general development agenda” could be two separate questions, [1] “The program aligns with health strategic plans” and [2] “The program aligns with the general development agenda”. Alternatively, the questions could be modified to make them clearer.

### Limitations

There are some limitations to the findings of this study. First, most of the programs in this study are in low- and middle-income countries and the findings in this study might not be applicable to programs in high-income countries. Secondly, large research-based pharmaceutical companies implemented the programs in this study and the findings might not be applicable to programs implemented by smaller pharmaceutical companies. Furthermore, we could only assess publicly available program information and could not assess whether companies had other program information requested in the WHO checklist that were not publicly available. Currently, most companies instead of independent third parties are providing data on their access programs. Third party verification of such program information could enhance its veracity but is currently not practiced in financial reporting as well as in many other private sector reporting activities.

## Conclusion

The WHO checklist provides a useful tool for assessing pharmaceutical industry led programs as most of checklist questions ask for information that should be available to those leading the program. Further revisions of the WHO checklist will help in refining it to improve clarity and content validity. The pilot testing of the checklist showed the existing gaps in the public reporting of program information such as paucity of information on additional resources required from or contributed by government and other stakeholders, program needs assessment, program alignment with country plans and capacity, detailed program monitoring and evaluation plan, risks mitigation strategies and sustainability plans.

## Supplementary information

**Additional file 1.** List of programs used to evaluate checklist.

## References

[1] Rockers PC, Wirtz VJ, Umeh CA, Swamy PM, Laing RO (2017). Industry-led access-to-medicines programs in low-and middle-income countries: strategies and evidence. Health Aff.

[2] Fürst M, Sturchio JL, Kickbusch I, Galambos L (2019). Reframing the pharmaceutical sector contribution to access to medicines and universal health coverage: a business ethics perspective. The road to universal health coverage: innovation, equity, and the new health economy.

[3] Nusser H, Sturchio JL, Kickbusch I, Galambos L (2019). Novartis social business: a novel approach to expanding health care in developing countries. The road to universal health coverage: innovation, equity, and the new health economy.

[4] Clarke D, Doerr S, Hunter M, Schmets G, Soucat A, Paviza A. The private sector and universal health coverage, 2019. Bull World Health Organ. 97:434–5. 10.2471/BLT.18.225540. 10.2471/BLT.18.225540 PMC6560377 31210681

[5] World Health Organization (2017). Responding to industry programs to increase access to medicines and other health technologies in countries. EMP policy brief series no. 2.0.

[6] Asante AD, Zwi AB (2007). Public-private partnerships and global health equity: prospects and challenges. Indian J Med Ethics.

[7] Buse K, Walt G. The World Health Organization and global public-private health partnerships: in search of ‘good’ Global Health governance. In: Reich MR, editor. Public-private partnerships for public health. Harvard series on population and international health. 2002:1–8. Accessed December 31, 2018 from https://cdn1.sph.harvard.edu/wp-content/uploads/sites/480/2012/09/Partnerships_book.pdf.

[8] World Health Organization, 2019. Accountability. Accessed January 14, 2020 from https://www.who.int/health-laws/topics/governance-accountability/en/.

[9] Brinkerhoff DW (2004). Accountability and health systems: towards conceptual clarity and policy relevance. Health Policy Plan.

[10] O'Malley P, Rainford J, Thompson A (2009). Transparency during public health emergencies: from rhetoric to reality. Bull World Health Organ.

[11] Access Observatory. Accessed February 19, 2019 from https://www.accessobservatory.org/.

[12] Access to Medicine Index 2018. Methodology report. Accessed September 14, 2018 from https://accesstomedicineindex.org/media/atmi/2017-Methodology-2018-Access-to-Medicine-Index.pdf.

[13] IFPMA Partnerships Directory. Accessed February 13, 2019 from http://partnerships.ifpma.org/pages/.

[14] Access Accelerated Open Platform. Accessed February 19, 2019 from https://aaopenplatform.accessaccelerated.org/#!/layouts/home.html.

[15] Hooper PD, Greenall A (2005). Exploring the potential for environmental performance benchmarking in the airline sector. Benchmarking.

[16] Global Reporting. Reporting on community impacts. In: A survey conducted by the global reporting initiative: The University of Hong Kong and CSR Asia; 2008. Accessed January 14, 2020 from https://www.globalreporting.org/resourcelibrary/Reporting-on-Community-Impacts.pdf.

[17] de Grosbois D (2012). Corporate social responsibility reporting by the global hotel industry: commitment, initiatives and performance. Int J Hosp Manag.

[18] Rockers PC, Reich MR, Kettler H, Wirtz VJ (2018). Commitment to impact: strengthening measurement of industry-led access-to-medicines programs. Health Syst Reform.

[19] Schell SF, Luke DA, Schooley MW, Elliott MB, Herbers SH, Mueller NB (2013). Public health program capacity for sustainability: a new framework. Implement Sci.

[20] Blasinsky M, Goldman HH, Unützer J (2006). Project IMPACT: a report on barriers and facilitators to sustainability. Adm Policy Ment Health Ment Health Serv Res.

[21] Scheirer MA (2005). Is sustainability possible? A review and commentary on empirical studies of program sustainability. Am J Eval.

[22] Wright J, Williams R, Wilkinson JR (1998). Health needs assessment: development and importance of health needs assessment. BMJ.

[23] Buse K, Walt G (2000). Global public-private partnerships: part II-what are the health issues for global governance?. Bull World Health Organ.

[24] Kostyak L, Shaw DM, Elger B, Annaheim B (2017). A means of improving public health in low-and middle-income countries? Benefits and challenges of international public–private partnerships. Public Health.

[25] Walt G, Buse K (2000). Partnership and fragmentation in international health: threat or opportunity?. Tropical Med Int Health.

[26] Shediac-Rizkallah MC, Bone LR (1998). Planning for the sustainability of community-based health programs: conceptual frameworks and future directions for research, practice and policy. Health Educ Res.

[27] Marmot M (2007). Commission on social determinants of health. Achieving health equity: from root causes to fair outcomes. Lancet.

[28] United Nations sustainable development goals. Accessed August 23, 2018 from https://www.un.org/sustainabledevelopment/inequality/.

[29] Rockers PC, Laing RO, Ashigbie PG, Onyango MA, Mukiira CK, Wirtz VJ (2019). Effect of Novartis access on availability and price of non-communicable disease medicines in Kenya: a cluster-randomised controlled trial. Lancet Glob Health.

[30] Sinkowitz-Cochran RL (2013). Survey design: to ask or not to ask? That is the question. Clin Infect Dis.

[31] Olson K, Lavrakas PJ (2008). 2013. ‘Double-barreled question (online encyclopedia entry). Encyclopedia of survey research methods.

