# Supplementary material for: Public reporting on pharmaceutical industry-led access programs: alignment with the WHO medicine programs evaluation checklist
Source: J Pharm Policy Pract. 2020 Mar 26;13:5. doi: 10.1186/s40545-020-0204-z (PMC7098075; doi:10.1186/s40545-020-0204-z)
Supplement: Supplementary file 1 — Additional file 1. List of programs used to evaluate checklist. [file 40545_2020_204_MOESM1_ESM.docx]

# Appendix 1

List of programs used to evaluate checklist

| Company | Program | Start date | Countries | Disease focus | Program description | Medicines/ vaccines included |
| --- | --- | --- | --- | --- | --- | --- |
| AbbVie | Advancing toward Universal Health Coverage in Kenya (Perpetual Home-Based Counseling and Testing Program in Kenya) | 2007 | Kenya | Non-communicable Diseases e.g. diabetes, hypertension; Women’s and Child Health; HIV/AIDS; Tuberculosis; Soil-Transmitted Helminthiasis; General Health | Counselors offer door-to-door counseling, testing and linkage to care and treatment. | No |
| Astellas | Action on Fistula | 2014 | Kenya | Obstetric fistula | Identify and treat women with obstetric fistula as well as supporting those who receive the surgical intervention throughout their treatment journey. | No |
| Astra Zeneca | Healthy Heart Africa | 2014 | Ethiopia, Kenya, Tanzania | Hypertension | Raise awareness around lifestyle choice and CVD risk factors, train providers and  facilitate access to anti-hypertensives | No |
| Bayer | Fighting African Sleeping Sickness | 2002 | Angola  Cameroon  Central African Republic  Chad  Congo  Côte d'Ivoire  Democratic Republic of the Congo  Equatorial Guinea  Gabon  Gambia  Kenya  Malawi  Nigeria  Rwanda  South Africa  Uganda  Tanzania  Zambia  Zimbabwe | African Sleeping Sickness | Support the World Health Organization (WHO) eliminate Human African Sleeping Sickness by donating suramin for the treatment of trypanosoma brucei gambiense infected Human African Trypanosomiasis patients. | Yes |
| Boehringer Ingelheim | Health is Priceless | 2012 | Brazil | Asthma | The program provides free access to asthma medication for underserved patients. | Yes |
| Bristol-Myers Squibb | Delivering Hope | 2002 | China, India, Japan | Hepatitis | Increase hepatitis B and C awareness,  promote hepatitis prevention,  support training for healthcare workers, and fund operational research | No |
| Daiichi | Cultivating Healthcare Workers in China (Daiichi Sankyo partners with Plan International) | 2015 | China | Non-communicable diseases, women and children health | Train medical professionals in community healthcare | No |
| Eisai | Hope to Her | 2014 | India  Indonesia  Myanmar  Thailand  Philippines | Breast cancer | Improve medication affordability through intra-country differential pricing model | Yes |
| Eli Lilly | AMPATH and Lilly Partnership | 2002 | Kenya | Diabetes  Cardiovascular Diseases  Cancer  Mental & Neurological Disorders  General Noncommunicable Disease Care (Health System) | Eli Lilly donates medicines including insulin, mental health products and oncolytics to AMPATH to provide treatment to people. Support AMPATH to provide screening for cancer and other chronic illnesses | Yes |
| Gilead | Frontline of Communities in the U.S (FOCUS) Program | 2010 | United States | Hepatitis B, Hepatitis C, HIV | An initiative to encourage routine HBV, HCV and HIV screening in healthcare settings | No |
| GlaxoSmithKline | Personal Hygiene and Sanitation Education (PHASE) Program | 1998 | Bangladesh, Bolivia, Brazil, India, Indonesia, Kenya, Malawi, Mexico, Nicaragua, Peru, Philippines, Senegal, Tajikistan, Uganda, UK and Zambia | Diarrhea | PHASE is an education program helping to reduce diarrhea-related disease by encouraging school children to wash their hands. | No |
| Johnson & Johnson | Mothers2mothers | 2001 | Ghana  Kenya  Lesotho  Malawi  Mozambique  Uganda  Zambia | HIV, Maternal and child health | mothers2mothers employs women living with HIV as Community Health Workers who work both at health facilities and door-to-door to educate and engage with women and families who have not received medical care or who have stopped treatment. | No |
| Merck & Co. | Access to Pediatric Formulations of Raltegravir | 2015 | Low- and middle-income countries | HIV | A licensing agreement with Medicines Patent Pool (MPP) for generic manufacturers to develop, manufacture and sell pediatric versions of raltegravir in low- and middle-income countries | Yes |
| Merck KGAa | Capacity Advancement Program | 2012 | Angola  Cameroon  Central African Republic  Congo  Côte d'Ivoire  Equatorial Guinea  Ethiopia  Ghana  Kenya  Liberia  Malawi  Mali  Mozambique  Nigeria  Rwanda  Senegal  Sierra Leone  South Africa  Uganda  United Republic of Tanzania  Zambia  Zimbabwe  United Arab Emirates  Bangladesh  India  Indonesia  Myanmar  Nepal  Sri Lanka  Cambodia | Non-communicable diseases | The program trains healthcare providers, medical students, and community health workers, conduct community awareness campaigns for diabetes, cancer and other NCDs and screen patients through medical camps | No |
| Novartis | Novartis Better Hearts Better Cities | 2017 | Brazil  Mongolia  Senegal | Cardiovascular Diseases | Improving cardiovascular health in low-income urban populations, through a multisector approach that addresses hypertension and its underlying risk factors | No |
| Novo Nordisk | Chronic care in humanitarian crises | 2018 | Global | Diabetes, hypertension | Supply low-cost human insulin in vials to the Red Cross operations globally. Start projects to provide care to hypertension and diabetes patients in humanitarian crises | Yes |
| Pfizer | Nay Bahay Safe Birthing Center Program | 2010 | Philippines | Maternal health | Construct lying-in maternal facilities that will give birthing mothers from indigent communities a safe place for labor with the supervision of licensed midwives | No |
| Roche | Socialised Pricing Programme in the Philippines | 2010 | Philippines | Cancer | A patient access program where qualified patients receive a discount or free cancer medication, based on their financial status. | Yes |
| Sanofi | Sanofi Dengue Control | 2010 | Brunei, Cambodia, Indonesia, Lao PDR, Malaysia, Myanmar, Philippines, Singapore, Thailand, Vietnam | Dengue | Research and development of dengue vaccine | Yes |
| Takeda | Palliative Care Training in sub-Saharan Africa | 2017 | Kenya | Cancer | Refining and updating national palliative care training curriculum and developing and maintaining palliative care data management system | No |
